# Acrylamide Elimination by Lactic Acid Bacteria: Screening, Optimization, In Vitro Digestion and Mechanism

**DOI:** 10.3390/microorganisms10030557

**Published:** 2022-03-03

**Authors:** Amal S. Albedwawi, Reem Al Sakkaf, Ahmed Yusuf, Tareq M. Osaili, Anas Al-Nabulsi, Shao-Quan Liu, Giovanni Palmisano, Mutamed M. Ayyash

**Affiliations:** 1 Department of Food Science, College of Agriculture and Veterinary Medicine, United Arab Emirates University (UAEU), Al Ain P.O. Box 15551, United Arab Emirates; 201690240@uaeu.ac.ae; 2 Department of Chemical Engineering, Center for Membrane and Advanced Water Technology (CMAT), Research and Innovation on CO_2_ and Hydrogen (RICH), Khalifa University of Science and Technology, Abu Dhabi P.O. Box 127788, United Arab Emirates; reem.alsakkaf@ku.ac.ae (R.A.S.); ahmed.yusuf@ku.ac.ae (A.Y.); giovanni.palmisano@ku.ac.ae (G.P.); 3 Department Clinical Nutrition and Dietetics, University of Sharjah, Sharjah P.O. Box 27272, United Arab Emirates; tosaili@sharjah.ac.ae; 4 Department of Nutrition and Food Technology, Jordan University of Science and Technology, Irbid 22110, Jordan; anas_nabulsi@just.edu.jo; 5 Department of Food Science and Technology, Faculty of Science, National University of Singapore, Singapore 117542, Singapore; fstlsq@nus.edu.sg

**Keywords:** acrylamide, TEM, SEM-EDS, FTIR, Box-Behnken design, the reduction mechanism

## Abstract

Acrylamide is a toxic compound that is formed in cooked carbohydrate-rich food. Baking, roasting, frying, and grilling are cooking methods that cause its formation in the presence of reducing sugar and asparagine. To prevent acrylamide formation or to remove it after its formation, scientists have been trying to understand acrylamide formation pathways, and methods of prevention and removal. Therefore, this study aimed to: (1) screen newly isolated LAB for acrylamide removal, (2) optimize conditions (pH, temperature, time, salt) of the acrylamide removal for selected LAB isolates using Box-Behnken design (BBD), (3) investigate the acrylamide removal abilities of selected LAB isolates under the in vitro digestion conditions using INFO-GEST2.0 model, and (4) explore the mechanism of the acrylamide removal using scanning electron microscopy coupled with energy-dispersive X-ray spectroscopy (SEM-EDS), zeta potential, transmission electron microscopy (TEM) measurement, and Fourier transform infrared spectroscopy (FTIR). Forty strains were tested in MRS broth, where *Streptococcus lutetiensis* and *Lactiplantibacillus plantarum* had the highest capability of acrylamide removal by 39% and 26%, respectively. To enhance the binding ability, both strains were tested under controlled conditions of pH (4.5, 5.5 and 6.5), temperature (32 °C, 37 °C and 42 °C), time (14, 18 and 22 h), and NaCl (0%, 1.5% and 3% *w*/*v*) using Box-Behnken design (BBD). Both strains removed more acrylamide in the range of 35–46% for *S. lutetiensis* and 45–55% for *L. plantarum*. After testing the bacterial binding ability, both strains were exposed to a simulated gastrointestinal tract environment, removing more than 30% of acrylamide at the gastric stage and around 40% at the intestinal stage. To understand the mechanism of removal, LAB cells were characterized via scanning electron microscopy coupled with energy-dispersive X-ray spectroscopy (SEM-EDS) and transmission electron microscopy (TEM) techniques. Cell charges were characterized by zeta potential and functional groups analyzed by Fourier transform infrared spectroscopy (FTIR). Results indicated that increasing cell wall thickness improved acrylamide adsorption capacity. Both FTIR and EDS indicated that functional groups C=O, C-O, and N-H were associated with acrylamide adsorption.

## 1. Introduction

Acrylamide is a chemical compound that is formed in heated carbohydrate-rich food. It is known to be a neurotoxin carcinogen in animals and a probable carcinogen in humans [1]. In addition, toxicological studies have provided proof that acrylamide can be genotoxic, neurotoxic, and toxic to the reproductive system [2]. After the discovery of acrylamide formation in food during the Maillard reaction with the presence of reducing sugar and free asparagine [3], both the Food and Drug Organization/World Health Organization and European Food Safety Authority started scanning the products in the markets and published guidelines to minimize acrylamide risks [4,5].

French fries, roasted coffee, potato chips, bread, pastries, breakfast cereals, and biscuits are some of the food types that might increase acrylamide intake [6]. Acrylamide is an unsaturated amide that can enter the body through food ingestion, inhalation of acrylamide that pollutes air, and skin contact. It is odorless, colorless, and highly soluble in water [2]. Since the discovery of acrylamide’s risks and its occurrences in food, scientists have published studies on factors (e.g., pH, temperature, NaCl) and measures (e.g., changes in raw materials, changes in cooking or processing conditions, or cooking with the emerging techniques such as irradiation, ultrasound, microwave) to control and reduce acrylamide formation [7].

Several attempts have been made to mitigate acrylamide presence in food by reducing its formation or removing formed acrylamide [7]. For example, it has been reported that additions of salt (NaCl), grape polyphenols extract, and *p*-coumaric acid-phosphate buffer to biscuits, potato chips, and fried potatoes, respectively, decreased acrylamide formation but changed the sensory characteristics of food [8,9,10]. Manipulating the food processing conditions (temperature, pH, incubation time, vacuuming) to reduce acrylamide formation has also been reported [11].

A microbial approach has been presented to mitigate acrylamide presence [7,12]. Lactic acid bacteria *Lacticaseibacillus casei* was used to ferment mixed rye and removed up to 20.2% of acrylamide [13]. Other strains such as *Pediococcus acidilactici* in biscuit, *Lactobacillus delbrueckii* in fried potato and *Lactobacillus casei* Shirota in potato chips were employed to remove acrylamide in these products. the percentages of the acrylamide removal were 78%, 51%, and 65–73%, respectively [14,15,16]. Lactobacillus is a Gram-positive, fermentative, facultatively anaerobic and non-sporeforming microorganisms. The family of Lactobacillaceae contains Lactobacillus, Paralactobacillus and Pediococcus genera [17].

The mechanism of acrylamide removal by microorganisms is not fully explored and understood [7]. The investigation of acrylamide removal by new microorganisms (such as lactic acid bacteria (LAB) and yeasts) is still highly sought, as suggested by various studies [7,18]. Moreover, it is crucial to investigate the acrylamide removal conditions (pH, temperature, water activity) surrounding the microorganisms. To the best of our knowledge, no information is available regarding acrylamide removal by LAB under the in vitro digestion condition.

Therefore, this study aimed to: (1) screen newly isolated LAB for acrylamide removal, (2) optimize conditions (pH, temperature, time, salt) of the acrylamide removal for selected LAB isolates using Box–Behnken design (BBD), (3) investigate the acrylamide removal abilities of selected LAB isolates under the in vitro digestion conditions using the INFOGEST2.0 model, and (4) explore the mechanism of the acrylamide removal using scanning electron microscopy coupled with energy-dispersive X-ray spectroscopy (SEM-EDS), zeta potential, transmission electron microscopy (TEM) measurement, and Fourier transform infrared spectroscopy (FTIR).

## 2. Materials and Methods

All chemicals were purchased from Sigma-Aldrich (St. Louis, MO, USA) unless otherwise is mentioned.

### 2.1. Strains of Bacteria

Forty strains of LAB previously isolated from food products in our laboratory [19,20,21] were assessed for their ability to eliminate acrylamide. All isolates were kept at −20 °C in 50% glycerol as a stock. These isolates were identified and classified under the following genera: (1) *Enterococcus*; (2) *Pediococcus*; (3) *Bifidobacterium*; (4) *Lactobacillus*; and (5) *Streptococcus*. All cultures were activated twice in de Man, Rogosa, and Sharpe (MRS) broth (LAB-M, Neogen Culture Media, Heywood, UK) by inoculating a loopful of the bacterial stock into 10 mL of MRS broth followed by incubation at 37 °C for 20 h.

### 2.2. Preparation of Stock and Working Solutions of Acrylamide

An acrylamide stock solution was prepared by dissolving 50 mg of powdered acrylamide (Sigma Chemical Co., St. Louis, MO, USA) in deionized water using a 50 mL volumetric flask to obtain a concentration of 1 mg/mL. Two working solutions were prepared by diluting the stock solution to obtain 50 µg/mL and 100 µg/mL in 10 mL of MRS broth in the screening stage.

### 2.3. Acrylamide Binding Assay—Preliminary Screening for Media Components and Bacterial Cultures

Ten milliliters of sterilized MRS broth supplemented with acrylamide at concentrations of 50 µg/mL or 100 µg/mL were inoculated with 1% of an activated culture followed by incubation at 37 °C for 20 h. Afterward, bacterial enumeration was performed by a pour-plate method using MRS agar followed by anaerobic incubation at 37 °C for 20 h. To prepare the samples for acrylamide analysis by LC-MS-MS, bacterial cells were removed by centrifugation (10,000× *g*, 10 min). The supernatants were collected for acrylamide analysis. Each sample was analyzed in duplicate, and for each group of samples, there were controls of MRS broth, MRS with bacteria, and MRS spiked with acrylamide.

### 2.4. Optimization of Acrylamide Removal Using Box–Behnken Design

Box–Behnken design (BBD) was employed to optimize the four variables at three levels each: pH (4.5, 5.5 and 6.5) using 1.0 N HCl or 1.0 M NaOH, temperature (32 °C, 37 °C and 42 °C) using anaerobic incubators, incubation period (14, 18 and 22 h), NaCl (0%, 1.5 and 3% *w*/*v*). Minitab v.21 was used to construct the experimental design and perform statistical analysis. The four independent variables were investigated with 27 experimental runs and three repetitive central points. The experiments were conducted for two different strains of LAB under aerobic and anaerobic conditions. Each experiment was carried out in triplicate, as presented in Table 1 and Table 2. The polynomial equation is presented as follows:

(1) $$Y={\;\beta }_{0}+\sum \beta_{i}X_{i}+\sum \beta_{\mathrm{ii}}X_{i}^{2}+\sum \beta_{\mathrm{ij}}X_{i}X_{j}$$

where Y is the predicted acrylamide concentration or bacterial count, and X_i_ and X_j_ are the independent variables. The studied variables were pH, temperature, time, and NaCl. β_o_ is the regression coefficient of the model and β_i_, β_ii_, and β_ij_ are the linear, quadratic and interaction coefficients, respectively. To investigate the relationship between the independent variables and the responses, two-dimensional response surface plots were constructed. The corresponding *p*-values from the ANOVA results were used to evaluate the significance.

The microbial population was measured by optical density at 600 nm with a spectrophotometer in 24-well plates using an Epoch 2 Microplate Spectrophotometer from BioTeck, Santa Clara, CA, USA. After the determination of bacterial populations, the samples were collected in 1.5 mL tubes and centrifuged at 10,000× *g* for 10 min. The supernatants were collected for further analysis for acrylamide using Agilent Technologies 6495 Triple Quad LC-MS-MS (Santa Clara, CA, USA).

### 2.5. In Vitro Digestion by INFOGEST2.0 Model

All samples of LAB strains were subjected to the in vitro gastrointestinal INFOGEST 2.0 protocol [22]. A 1 mL aliquot of the bacterial pellet suspension was subjected to in vitro digestion including oral phase (amylase 75 U/mL, salivary fluid SSF, 0.3 M CaCl_2_, 2 min, 37 °C), gastric phase (pepsin 2000 U/mL, gastric juice SGF pH 3.0, 0.3 M CaCl_2_, 120 min, 37 °C) without adding the rabbit gastric extract, and intestinal phase (pancreatin 100 U/mL, bile 10 mmol/L, duodenal juice SIF pH 7.0, 0.3 M CaCl_2_, 120 min, 37 °C). Continuous shaking at 120 rpm was employed during the in vitro digestion. During the process of INFOGEST 2.0, samples were collected for both bacterial count and acrylamide analysis. Serial dilution was used to measure the bacterial count directly after taking the samples. For acrylamide analysis, samples were kept frozen under −20 °C until further analysis.

### 2.6. Quantification of Acrylamide by LC-MS-MS

The amount of acrylamide present in the aqueous fraction was determined by using the Agilent 1290 Infinity LC system equipped with MS/MS detector (Agilent, Santa Clara, CA, USA) using Column Hypercarb C_18_ (2.1 × 100 mm, 5.0 μm, Thermo Scientific, Waltham, MA, USA) with a mobile phase of 1% acetic acid in deionized water with a flow rate of 0.2 mL/min, an injection volume of 20 μL and a column temperature of 35 °C. An external acrylamide standard curve of 0, 5, 10, 25, 50, 75, 100, 125 and 150 μg/mL was constructed for quantification.

### 2.7. Understanding Mechanism of Acrylamide Binding by LAB

#### 2.7.1. Preparation of Samples and Binding Assay

The two strains of the LAB were activated twice in MRS broth for 24 h under 37 °C. An aliquot of the activated cultures from the second subculture’s culture to obtain 10^9^ cfu/mL of the second subculture was added at 1% *v*/*v* to 10 mL of fresh MRS broth containing 10 µg/mL acrylamide and incubated under the following conditions: 37 °C, 0% NaCl and pH 6.5 for 18 h. After the incubation, samples were centrifuged under 5000× *g*, 10 min, at 4 °C. Supernatants were removed, and bacterial cells were collected in 0.1 M, pH 7.0 phosphate buffer with 1.5 mL tubes and kept under −20 °C until analysis.

#### 2.7.2. Transmission Electron Microscopy (TEM) Measurement

LAB cells were characterized by using Tecnai transmission electron microscopes (TEM) (FEI Company, Hillsboro, OR, USA) operating at 200 kV. When the samples were prepared (pre-fixed, rinsed, post-fixed, dehydrated, soaked, and embedded), they were cut into 50–60 nm thick sections by using Ultramicrotome-UC6 (Leica Microsystems GmbH, Wetzlar, Germany) [23].

#### 2.7.3. Estimation of Zeta Potential

The zeta potential of the LAB cells was measured to test the stability of a colloid. A micro-electrophoretic apparatus Zeta Plus (Zetasizer Nano ZS 90, Malvern Instruments Ltd., Worcestershire, UK) was used to determine zeta potential. The experiment was performed at room temperature and the pH was adjusted using 0.1 M NaOH and 0.1 M HCl [23].

#### 2.7.4. Fourier Transform Infrared Spectroscopy (FTIR) Analysis

FTIR analysis was conducted by attenuated total reflectance (ATR)-FTIR spectroscopy using a Spectrum Two IR coupled with a Universal ATR (UATR) unit (Perkin-Elmer Inc., Norwalk, CT, USA) to determine the functional groups and putative binding sites that would have an impact on acrylamide adsorption. Bacterial cell samples were freeze-dried and directly positioned on a Diamond/ZnSe crystal plate (Perkin-Elmer). The IR spectral range was 4000–400 cm^−1^ [23,24,25].

#### 2.7.5. Scanning Electron Microscopy Coupled with Energy-Dispersive X-ray Spectroscopy (SEM-EDS)

SEM-EDS was performed to understand the morphology and elementary composition of bacterial cells. The bacterial cells were tested after adding acrylamide to the samples and were fixed with 2.5% (*v*/*v*) glutaraldehyde with 1% osmium tetroxide. *S. lutetiensis* and *L. plantarum* radius, height, and elemental composition were evaluated by Quanta 250 ESEM [24,26]. A 5 uL amount of each sample was deposited on a piece of aluminum foil attached to the stainless-steel stub with carbon tape and allowed to dry before loading it into the SEM machine using a stub holder.

### 2.8. Statistical Analysis

To determine both mean values and standard deviations of results from screening of samples, Minitab v.21 (Minitab Ltd., Coventry, UK) was used. The BBD was performed, and the responses were analyzed using Minitab v.21.

## 3. Results

### 3.1. Screening of Acrylamide Removal by LAB

**Figure 1:**
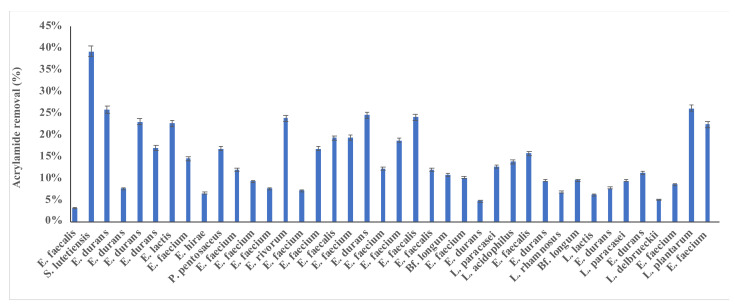
Acrylamide removal (%) of 40 newly isolated lactic acid bacteria.

Figure 1 was used to assess the acrylamide removal ability in the screening stage. The conventional screening was used to examine the capability of LAB in binding to acrylamide. Acrylamide removal ranged from 3.1% to 39.1% (see Figure 1). The strains *S. lutetiensis* (39%) and *L. plantarum* (26%) had the highest acrylamide removal. It has been reported that acrylamide removal by microorganisms is species- and strain-dependent [7]. These two strains were selected for further study. Both strains *Streptococcus lutetiensis* and *Lactiplantibacillus plantarum* were certified as Generally Recognized as Safe by the US Food and Drug Administration [27]. *Streptococcus lutetiensis* strains are isolated from bovine and *Lactiplantibacillus plantarum* isolated from fermented food and dairy products. *Streptococcus* is commonly added to dairy, soy, and vegetable products in the food industry. *Lactiplantibacillus plantarum* is used to ferment cheese and other products [27].

### 3.2. Optimization of Acrylamide Removal

**Figure 2:**
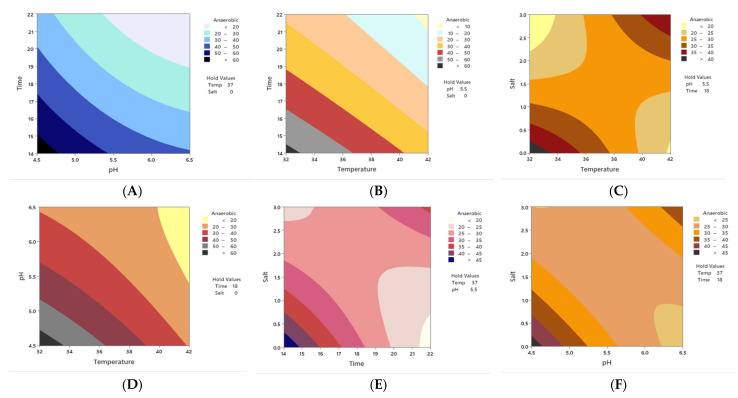

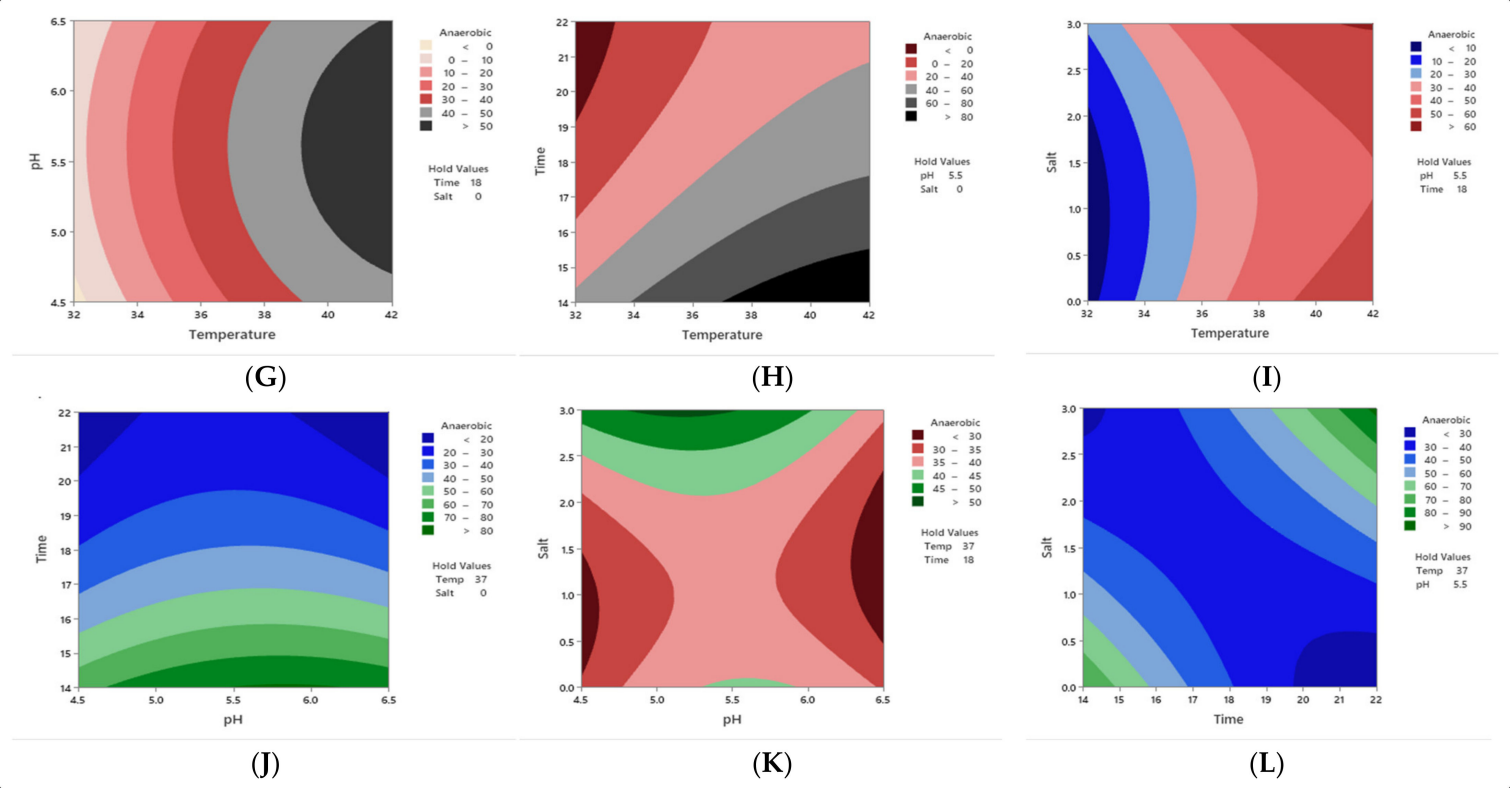
Contour plots of acrylamide removal in anaerobic conditions for *S. lutetiensis* (**A**–**F**) and *L. plantarum* (**G**–**L**) under controlled conditions of incubation time of 14–22 h, salt (NaCl) of 0.0–3.0%, and incubation temperature of 32–42 °C and pH of 4.5–6.5.

Table 1 presents the results of the optimization of acrylamide removal by both *S. lutetiensis* and *L. plantarum* using BBD. Table 2 presents the analysis of variance for both strains. Figure 2A–L shows contour plots of the results of running the experiments using BBD. Based on the tested runs’ results of *S. lutetiensis* and *L. plantarum* in Table 1, two regression equations are describing the true relationships between the responses and the independent variables for both strains as follows:

$$\begin{array}{ccc} Acrylamide\;removal\\ & = & 713-9.9X_{1}-101.6X_{3}-134.6X_{2}-11.8X_{4}-0.001X_{1}{}^{2}+4X_{3}{}^{2}+0.131X_{4}{}^{2}+1.62X_{2}{}^{2}\\ & + & 1.22X_{1}X_{3}+0.084X_{1}X_{4}+1.633X_{1}X_{2}+0.020X_{3}X_{4}+6.39X_{3}X_{2}+1.829X_{4}X_{2} \end{array}$$

(1) Regression equation in uncoded units of *L. plantarum*

$$\begin{array}{ccc} Acrylamide\;removal\\ & = & 830-19.5X_{1}-145X_{3}-134.6X_{2}-11.8X_{4}-0.001X_{1}{}^{2}+4X_{3}{}^{2}+0.131X_{4}{}^{2}+1.62X_{2}{}^{2}\\ & + & 1.22X_{1}X_{3}+0.084X_{1}X_{4}+1.633X_{1}X_{2}+0.020X_{3}X_{4}+6.39X_{3}X_{2}+1.829X_{4}X_{2} \end{array}$$

After running analysis of variance (ANOVA) presented in Table 2, the statistically significant results were determined by *F*-value = 2.31 and *p*-value was insignificant (*p* > 0.077) for *S. lutetiensis*, unlike *L. plantarum,* which showed statistically significant results of both *F*-value = 7.00 and *p*-value of (*p* < 0.001).

The coefficient of determination values R^2^ was 0.72 for all models and suggested that the developed models have the goodness of fit that could explain >72% of the total variation as presented in Supplementary Materials Table S1. The adjusted *R* ^2^ was 41.30% (Supplementary Materials Table S1) for *S. lutetiensis*. *L. plantarum* presented higher results and had the goodness of fit that could explain up to 89.09% of the total variation and the predicted *R* ^2^ value of 41.95%. The *p*-values for the lack of fit for the models were not significant for both strains (*p* > 0.335, *p* > 0.321) of *S. lutetiensis* and *L. plantarum*, respectively.

For *S. lutetiensis*, Figure 2A–F shows the effect of salt and temperature at pH of 5.5 and incubation time 18 h, where acrylamide was at its lowest level at NaCl of 0.0 and 32 °C, and this relationship is statistically significant (*p* < 0.01). When *S. lutetiensis* was incubated under aerobic conditions, the bacterium showed different behaviors. The binding ability of the bacterium under the anaerobic conditions was better, by comparing Figure 1 and Figure 2A–F.

There were no significant relationships between pH, temperature, salt, and incubation time (Table 2). *S. lutetiensis* bound higher acrylamide amounts under anaerobic conditions. The rest of the factors were not significant, nor were their interactions (Table 2). By examining Figure 1, namely pH and temperature at 18 h and salt (0.0), acrylamide was at its lowest level when the pH was at 4.5 and the temperature was at 32 °C. By analyzing Figure 2A–F and studying the interaction of the factors with one another and their impact on *S. lutetiensis* binding abilities, the optimum conditions were: NaCl (0.0), time (14–18), pH (4.5–5.5), and temperature (32–37 °C), where acrylamide results were at their minimum levels.

The only significant relationship for *L. plantarum* was for time and salt *p* < 0.01) (Table 2). Figure 2L shows the incubation time of 14–22 h and salt of 0.0–3.0% under controlled conditions of pH (5.5) and temperature (37 °C), the highest reduction of acrylamide was obtained under incubation time of 14 and 22 h and salt of 0 and 3%.

### 3.3. Acrylamide Removal Under In Vitro Digestion

The percentages of acrylamide removal were 35.1 ± 1.22% and 41.2 ± 2.32% for *L. plantarum* and *S. lutetiensis*, respectively. This indicates that acrylamide removal is species-dependent. Our results imply that LAB could remove the formed acrylamide during in vitro digestion. To the best of our knowledge, this is the first report on acrylamide removal by LAB during in vitro digestion. The effect of the matrix on acrylamide removal during in vitro digestion was also investigated. Our results showed that the media matrix (the in vitro digestion solutions) had a minor effect (<1.1%) on acrylamide removal.

### 3.4. Mechanisms of Acrylamide Removal

#### 3.4.1. Estimation of Zeta Potential of Bacterial Cells

The average zeta potentials of *S. lutetiensis* and *L. plantarum* suspensions were −0.72 and 0.37 mV, respectively. Both strains showed excellent to maximum coagulation results, and both had high adsorption rates. The control sample, which had the bacteria without acrylamide, had a zeta potential of −13.51 mV, which suggests that adding acrylamide to the bacterial samples might have had an impact on the zeta potential. Noting that pH is a major factor that affects zeta potential, it might be suggested that acrylamide changed the pH of the samples.

#### 3.4.2. Fourier Transform Infrared Spectroscopy (FTIR) Analysis

**Figure 3:**
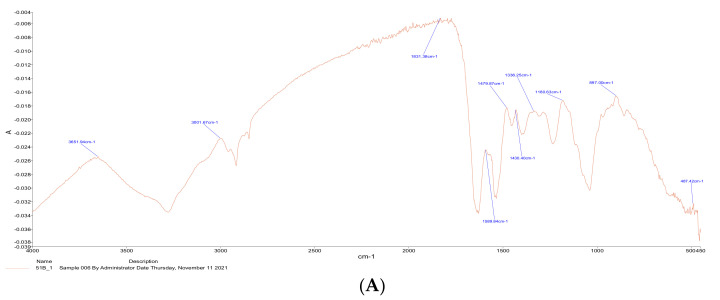

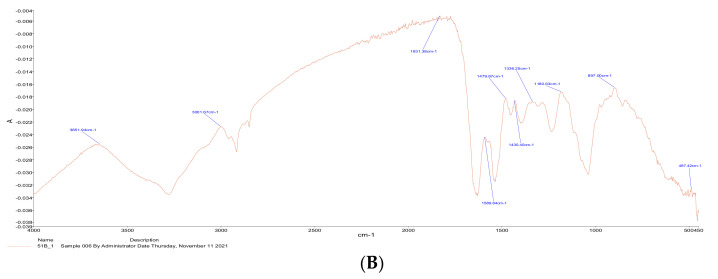
FTIR spectra of *S. lutetiensis* (**A**) and *L. plantarum* (**B**) label for (**A**,**B**).

The FTIR spectra of *S. lutetiensis* and *L. plantarum* are presented in Figure 3A,B. The two strains showed different peaks comparing them to each other. The different peaks refer to the differences in the functional groups (C-O, C=O, and N-H), which might lead to the variations in adsorption capacities.

#### 3.4.3. Scanning Electron Microscopy Coupled with Energy-Dispersive X-ray Spectroscopy (SEM-EDS)

**Figure 4:**
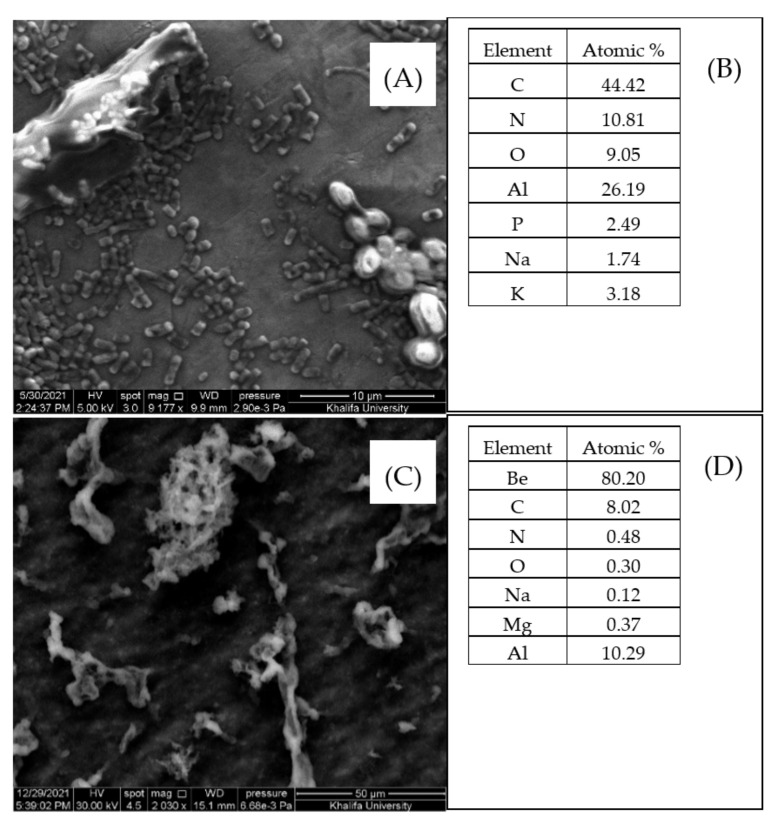
SEM-EDS images and elements of *S. lutetiensis* (**A**,**B**) and *L. plantarum* (**C**,**D**) and the peaks of the EDS of the main elements.

Figure 4A–F presents the elements of *S. lutetiensis* and *L. plantarum*. Figure 4C shows that the most dominant elements in *S. lutetiensis* were C, N, O, Al, P, Na, and K, whereas *L. plantarum* had Be, C, N, O, Na, Mg, and Al in different atomic percentages. The control did not show atomic percentages of O, Na, Mg, Al, or K, which might explain that acrylamide caused some changes in the chemical composition of the bacteria. *S. lutetiensis* and *L. plantarum* had significant differences in the composition of elements. Interestingly, Beryllium (Be) was found in a very high amount in *L. plantarum* and P and K lower than 0.05 atomic % (Figure 4C,F). The existence of the three atoms oxygen (O), nitrogen (N), and carbon (C) might also explain acrylamide adsorption because, in the case of the control, oxygen was zero.

#### 3.4.4. Transmission Electron Microscopy (TEM) Measurement

**Figure 5:**
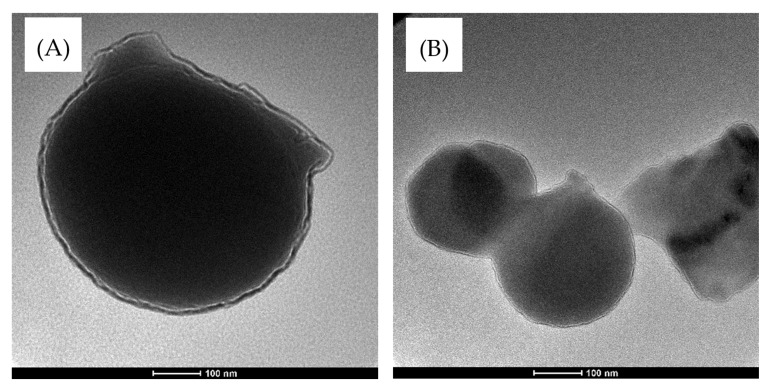
TEM of (**A**) *S. lutetiensis* and (**B**) *L. plantarum* showing the shape of the cells and the thickness of the cell walls.

The TEM images of *S. lutetiensis* and *L. plantarum* are displayed in Figure 5. Based on the visual observations, the TEM images showed an increase in the cell wall thickness compared with the bacterial cells grown in MRS without acrylamide. This test was done for qualitative purposes and not for measuring the cell wall thickness.

## 4. Discussion

Several new approaches have been employed by scientific researchers to reduce acrylamide and other toxins in food, at the industrial scale, and maintain the quality of the final products. Some LAB strains showed positive results. This research analyzed the ability of two strains out of forty. The latest studies published on LAB’s ability to reduce acrylamide and toxins suggest that the following mechanisms are involved: adsorption, degradation, precursor reduction, or antioxidant properties that reduce the accumulation of amines and N-nitrosamines [2].

It is reported that *Limosilactobacillus reuteri* and *Lacticaseibacillus casei Shirota* could remove up to 24.01% and 24.95% of acrylamide, respectively, after 12 h of incubation in MRS broth. *L. reuteri* NRRL 14171 and *L. casei Shirota* had the highest binding capacity of acrylamide in a study of fourteen LAB [28]. All strains exhibited high stability after repeated washing and were pH-dependent [28]. However, the current study revealed that under an uncontrolled environment, both *S. lutetiensis* and *L. plantarum* removed 39% and 26% of acrylamide, respectively, while under controlled conditions the removal percentage increased up to 46% and 57%, respectively.

LAB can also bind other toxins. For example, *Lactobacillus johnsonji* CECT 289 had the highest reduction of 97.4% removal of ochratoxin A in MRS among all the tested strains of LAB under gastrointestinal digestion [29]. In addition, strains of *L. plantarum* removed up to 47.80% of zearalenone [30]. The removal is affected by several factors like bacterial cells density, the concentration of the toxin, the viability of the bacteria, and incubation temperature. *L. casei* was found to have the ability to remove up to 49.2% of aflatoxin and tolerate bile salts [31]. *L. plantarum* had a significant binding ability with cadmium [32]. It also exhibited good anti-oxidative properties and resistance to simulated gastrointestinal conditions [32]. *L. casei Shirota* also exhibit good binding ability to remove up to 70% of acrylamide under different simulated gastrointestinal conditions [16]. These results indicate that adsorption capacity is strain- and bacterial count-dependent, which is in agreement with the results obtained herein [23,24]. It was recognized that the cell wall had a major role in the adsorption of acrylamide by LAB [23,24]. The present study indicated that cell wall components are crucial in acrylamide adsorption. SEM-EDS results showed significant changes in the wavenumbers of the C-O, C=O, and N-H, which are the functional groups in the LAB cell wall affecting acrylamide adsorption [33]. The present study demonstrated that LAB could remove acrylamide during in vitro digestion. This implies that the presence of LAB in food provides an additional safety margin against acrylamide. To the best of our knowledge, this study is the first attempt to investigate acrylamide removal by LAB under simulated GIT conditions (in vitro).

## 5. Conclusions

Overheating foods rich in carbohydrates might result in the formation of acrylamide, which is a carcinogenic chemical compound. Forty strains of lactic acid bacteria showed different acrylamide-binding abilities. Both *S. lutetiensis* and *L. plantarum* had the highest capability of acrylamide removal by 39% and 26%, respectively. In a simulated intestinal tract system, both strains removed more than 30–40% of the acrylamide.

To understand the mechanism of acrylamide removal, lactic acid bacteria cells were characterized via scanning electron microscopy coupled with energy-dispersive X-ray spectroscopy (SEM-EDS) and transmission electron microscopy (TEM) techniques. Cell charges were characterized by zeta potential and functional groups by Fourier transform infrared spectroscopy (FTIR). Results indicated that increasing cell wall thickness improves acrylamide adsorption capacity. Both FTIR and EDS indicated that functional groups C=O, C-O, and N-H are associated with acrylamide adsorption. The results of both strains indicate that LAB can be used to eliminate acrylamide in gastrointestinal systems, but further studies are needed in vivo in the human being gastrointestinal system.

## Figures and Tables

**Table 1:** Box–Behnken experimental design with coded variables and the responses of acrylamide removal (%) for *S. lutetiensis* and *L. plantarum* under anaerobic condition.

Runs	Temperature (°C) (X1)	pH (X2)	Time (h) (X3)	NaCl (g/100 mL) (X4)	(%) Growth	
					*S. lutetiensis*	*L. plantarum*
1	42	6.5	18	0.0	17.6	52.9
2	42	5.5	18	1.5	35.1	53.7
3	42	5.5	14	1.5	24.8	56.2
4	37	6.5	18	1.5	21.4	8.0
5	37	5.5	22	0.0	12.9	15.5
6	32	5.5	22	1.5	25.7	16.8
7	37	4.5	14	3.0	24.4	20.0
8	37	4.5	18	1.5	35.8	24.7
9	42	5.5	18	1.5	28.8	54.0
10	37	5.5	18	0.0	27.6	34.6
11	37	5.5	14	1.5	33.6	45.0
12	32	6.5	18	3.0	21.8	8.3
13	37	6.5	22	1.5	24.9	42.5
14	37	6.5	14	1.5	35.0	39.7
15	37	5.5	18	0.0	33.6	43.0
16	32	5.5	18	0.0	46.5	7.9
17	37	6.5	18	0.0	27.5	41.0
18	37	5.5	14	3.0	25.4	35.5
19	42	4.5	18	3.0	27.3	55.5
20	32	5.5	14	1.5	33.6	9.3
21	37	4.5	18	1.5	30.0	38.5
22	37	5.5	22	1.5	24.6	47.0
23	32	4.5	18	3.0	15.8	14.7
24	37	5.5	18	3.0	27.6	50.6
25	37	4.5	22	1.5	32.7	51.4
26	32	5.5	18	1.5	20.1	12.9
27	42	5.5	22	1.5	23.6	56.8

**Table 2:** Analysis of variance for *S. lutetiensis* and *L. plantarum.*

Source	*S. lutetiensis*			*L. plantarum*	
	DF	*F*-Value	*p*-Value	*F*-Value	*p*-Value
Model	14	2.31	0.077	7.00	0.001
Linear	4	1.89	0.177	15.96	0.000
Temp	1	0.01	0.911	62.51	0.000
pH	1	1.59	0.232	0.34	0.569
Time	1	6.97	0.022	0.39	0.546
Salt	1	0.65	0.437	1.84	0.200
Square	4	0.62	0.658	3.30	0.048
Temp × Temperature	1	0.01	0.922	4.86	0.048
pH × pH	1	1.51	0.243	2.63	0.131
Time × Time	1	0.45	0.513	4.42	0.057
Salt × Salt	1	1.03	0.330	2.55	0.136
2-Way Interaction	6	3.15	0.043	3.07	0.047
Temperature × pH	1	1.45	0.251	0.00	0.988
Temperature × Time	1	0.38	0.550	0.16	0.695
Temperature × Salt	1	9.07	0.011	0.18	0.678
pH × Time	1	0.00	0.980	0.59	0.457
pH × Salt	1	4.11	0.065	0.25	0.627
Time × Salt	1	4.17	0.064	13.49	0.003
Error	12				
Lack-of-Fit	9	1.84	0.335	1.92	0.321
Pure Error	3				
Total	26				

## Data Availability

Not applicable.

## Supplementary Materials

The following supporting information can be downloaded at: https://www.mdpi.com/article/10.3390/microorganisms10030557/s1, Table S1. Model Summary for Streptococcus lutetiensis and Lactobacillus plantarum.
