# Supplementary material for: Acrylamide Elimination by Lactic Acid Bacteria: Screening, Optimization, In Vitro Digestion and Mechanism
Source: Microorganisms. 2022 Mar 3;10(3):557. doi: 10.3390/microorganisms10030557 (PMC8953158; doi:10.3390/microorganisms10030557)
Supplement: Supplementary file 1 [file microorganisms-10-00557-s001.zip › microorganisms-1616130-supplementary.pdf]

microorganisms

Supplementary materials

Table S1. Model Summary for Streptococcus lutetiensis and Lactobacillus plantarum.

	S	R-sq	R-sq(adj)	R-sq(pred)
Streptococcus lutetiensis	5.45316	72.91%	41.30%	0.00%
Lactobacillus plantarum	8.58303	89.09%	76.35%	41.95%
